# Does recall time matter in verbal autopsies? Evidence from urban informal settlements in Nairobi, Kenya

**DOI:** 10.12688/wellcomeopenres.16243.2

**Published:** 2021-03-01

**Authors:** Donnie Mategula, Judy Gichuki

**Affiliations:** 1 Malawi-Liverpool-Wellcome Trust Clinical Research Programme , P.O Box 30096, Blantyre, Malawi; 2 Nairobi City County Government, Health Services Department, P.O. Box 34349 -00100, Nairobi, Kenya

**Keywords:** Nairobi, Verbal autopsy, Cause of death, Recall, time

## Abstract

**Background:** To assign a cause of death to non-medically certified deaths, verbal autopsies (VAs) are widely used to determine the cause of death. The time difference between the death and the VA interview, also referred to as recall time, varies depending on social and operational factors surrounding the death. We investigated the effect of recall time on the assignment of causes of death by VA.

**Methods: **This is a secondary analysis of 2002-2015 survey data of the Nairobi Urban Health Demographic Surveillance System (NUHDSS). The independent variable recall time was derived from the date of death and the date when the VA was conducted. Univariate and multivariate logistic regression methods were used to calculate odds ratios of assigning a cause of death in defined categories of recall time.

**Results: **There were 6218 deaths followed up between 2002 and 2016, out of which 5495 (88.3%) had VAs done. Recall time varied from 1-3001 days (median  92 days, IQR 44-169 days). Majority of the VAs (45.7%) were conducted between 1-3 months after death. The effect of recall time varied for different diseases. Compared to VAs conducted between 1-3 months, there was a 24% higher likelihood of identifying HIV/AIDS as the cause of death for VAs conducted 4-6 months after death (AOR 1.24; 95% CI 1.01-1.54; p-value = 0.043) and a 40% increased chance of identifying other infectious diseases as the cause of death for VAs conducted <1 month after death (AOR 1.4; 95% CI 1.02-1.92, p-value = 0.024).

**Conclusions:** Recall time affected the assignment of VA cause of death for HIV/AIDS, other infectious diseases,maternal/neonatal and indeterminate causes. Our analysis indicates that in the urban informal setting, VAs should be conducted from one month up to 6 months after the death to improve the probability of accurately assigning the cause of death.

## Introduction

Mortality data collected as part of vital registries, disease surveillance systems and epidemiological studies is essential for decision making ^1^. The need for its accuracy and reliability cannot be overemphasized ^2, 3^. Currently, the gold standard method for assigning the specific cause of death is through complete diagnostic autopsy ^4^ The alternative, where this is not possible, is certification by a medical practitioner using guidelines stipulated in the International classification of diseases and related health problems (ICD), currently available in the eleventh version ^5^

In countries where the vital registration systems are not fully developed, or in situations where there are challenges in medical certification of deaths, such as a home death, verbal autopsies (VAs) are an important means of assigning the cause of death ^6, 7^. The VA consists of an interview using standardized questionnaires with the deceased person’s close relatives or caregivers who is aware of the circumstances leading to the death ^7^. The most widely used VA questionnaires are by the World Health Organization ^7^ and the Population Health Metrics Research Consortium ^8^.

After completion of the VA interview, data from the VA questionnaire is then interpreted to assign the cause of death. Methods for assigning the cause of death from the VA interview data vary and include the use of either physician certified verbal autopsies(PCVA) or computer coded verbal autopsy (CCVA) systems that utilise algorithms, statistical techniques, machine learning and deep learning approaches ^9, 10^. A systematic review comparing PCVAs with various CCVA systems found that although the methods differed in the cause of death output, none of the VA interpretation methods reviewed was superior to the others ^9^. Use of CCVA may however decrease the time and cost associated with PCVAs and improve consistency and comparability ^1^.

A key element in the reliability of cause of death data as determined through VAs is the time between when the verbal autopsy was conducted and when the death occurred, also referred to as the recall time ^11^. There are varying optimal recall time to achieve maximum validity of a VA available in literature ranging from as soon as the death occurs up to 12 months and beyond ^12– 14^. Data from a study in South Asia indicates that the probability of assigning a correct cause of death by VA methods decreases by 0.55% per increasing month of the recall time ^11^. In contrast, a study in South Africa found that apart from neonatal causes, there was no impact on the validity of VA for recall time as long as one year ^15^. It is of importance that the timing of the VA not only takes account of the validity but also social and cultural factors ^16^. As such, it may be imperative to set context-specific optimal timing for VAs. In this paper, we aim to determine the optimal timing of conducting a VA to achieve higher odds of assigning the accurate cause of death in a low-income setting. We use mortality data collected from the Nairobi Urban Health Demographic Surveillance System (NUHDSS). This NUHDSS is run by the African population and research Centre (APHRC) in the informal settlements of Korogocho and Viwandani in Nairobi, Kenya ^17^

## Methods

### Study setting

This paper utilizes mortality data collected from two informal settlements in Nairobi Kenya, that form the NUHDSS, a DSS system run by APHRC. Korogocho is located in Ruaraka Sub-County in Nairobi and covers an area of 0.9 square km with a total population of 36,900 and a density of 42,401 persons per km ^2^ as per the 2019 Kenya population and housing census ^18^. Viwandani is located in Makadara Sub-County, it covers an area of 5 km ^2^ with a population of 43,070 and a density of 8554 persons per square km ^18^. Viwandani forms part of Nairobi’s industrial region and its inhabitants are mainly casual labourers within the industries in the area, while Korogocho is inhabited by residents mainly engaged within the informal job sector. Maps for the NUHDSS can be accessed elsewhere ^19^

Key challenges in Korogocho and Viwandani, as in many other urban informal settlements, include minimal formal infrastructure such as roads and piped water networks as well as rising cases of communicable and non-communicable diseases, rampant insecurity, environmental pollution, alcohol and drug abuse ^17^

### Design

This secondary analysis uses VA data collected through a series of surveys by the APHRC in the NUHDSS. The general methodology of the NUHDSS is summarized elsewhere ^20^. In summary, these were a series of surveys conducted from 2002 to 2015 using methodologies described by The International Network for the Demographic Evaluation of Populations and their Health (INDEPTH) network ^21^. Surveys were done every four months. Information on the events surrounding the death within the NUHDSS was collected by field interviewers and later independently validated by three physicians to assign a probable cause of death. Data from the questionnaire was then used as inputs in the InterVA-4 software, a tool that uses probabilistic models based on Bayes' theorem to interpret symptom data and determine possible causes of death ^22^. The output from the InterVA-4 model consists of up to three likelihoods per case attributed to different causes. An indeterminate residual portion is assigned when the sum of the probability of the three likelihoods is less than 100% ^22^. For this study, we utilized the output from the InterVA-4 model to carry out analysis of optimal recall time.

### Data analysis

We used STATA version 15.1 in the analysis and included all records where a successful VA was conducted. The main independent variable of interest in the analysis, recall time, is derived from the date of death and the date when the VA was conducted. Based on consideration of the average mourning time, and the acceptable time to conduct a VA as per the Kenya Verbal Autopsy Guidelines ^23^, we categorized recall time into less than 1 month, 1 to 3 months, 4 to 6 months, 7 to 12 months and greater than 12 months. The upper limit integer month for each category included all the possible decimals before the next category (for example, 1–3 months included all possible days from 1 month up to 3.99 months). In the descriptive analysis, we tabulate the recall time against other background characteristics to calculate their frequencies in the categories of recall time.

For each death, there were three possible causes as an output from the InterVA-4 model. The interVA-4 output was converted into cause-specific mortality fractions considering all the three probable causes of death assigned. The dependent variables were derived from each specific probable cause of death, transformed into a binary variable of yes/no. There were 22 most probable causes of death as an output in the interVA-4 model. To reduce data sparsity in our modelling, we collapsed these 22 probable causes of death into 12 categories guided by their counts and “reasonable relatedness”. Frequently occurring diseases such as tuberculosis and HIV/AIDS were retained in their original categories. Asthma, diabetes melitus, chronic obstructive pulmonary disease (COPD) and other chronic diseases were reclassified as other chronic/non communicable diseases. Injuries were grouped together with other external causes of death. Malaria and anaemia were grouped together given their limited numbers and that malaria is a major cause of anaemia in this region ^24^. Direct obstetric and neonatal causes were combined into one category as maternal/neonatal causes. Details of this re-categorization of the probable causes of death are found in Table 1. This approach to recategorization has also been applied in other studies ^15, 20^. 

**Table 1.** Cause of death recategorization criteria.

Cause of death as per interVA-4	Re-classified cause of death groups
HIV/AIDS	HIV/AIDS
Asthma	Other chronic/NCDs
Anaemia	Malaria/Anaemia
Cardiovascular	Cardiovascular
COPD	Other chronic/NCDs
Diabetes mellitus	Other chronic/NCDs
Diarrhoeal diseases	Other infectious diseases
Direct obstetric	Neonatal/Maternal
Infectious diseases	Other infectious diseases
Injuries	Injuries/Other external
Liver disease	Other chronic/ NCDs
Malaria	Malaria/Anemia
Malnutrition	Other chronic/NCDs
Meningitis	Meningitis
Neonatal causes	Neonatal/Maternal
Malignancies	Malignancies
Respiratory tract infections	Respiratory tract infections
Tuberculosis	Tuberculosis
Other communicable disease	Other infectious diseases
Other non-communicable disease	Other chronic/NCDs
Other external cause	Injuries/Other external
Indeterminate	Indeterminate

To investigate the effect of recall time on determining the cause of death outcome of the VA, logistic regression methods were used to calculate odds ratios of assigning a probable cause of death using the categories of recall time defined above. The 1 to 3 months recall time was used as the reference category for the logistic regression as the highest number of VAs were conducted within this period and this falls within the Kenya VA guidelines on appropriate time to conduct a VA. In the univariate logistic regression analysis, association of recall time and probable cause of death outcome of the VA was tested using chi square tests while in the multivariate analysis, likelihood ratio tests were carried out. Wald tests were used to determine the effect of various categories of recall time. The backward modelling approach was used in the multivariate analysis to adjust for the background characteristics.

### Ethical considerations

Permission to use the dataset was obtained from APHRC. The original NUHDSS study obtained ethical clearance from Kenya Medical Research Institute (KEMRI) Ethical Review Committee (Ref number KEMRI/RES/7/3/1)

## Results

### Distribution of deaths in the NUHDSS 2002–2016

**Figure 1.**
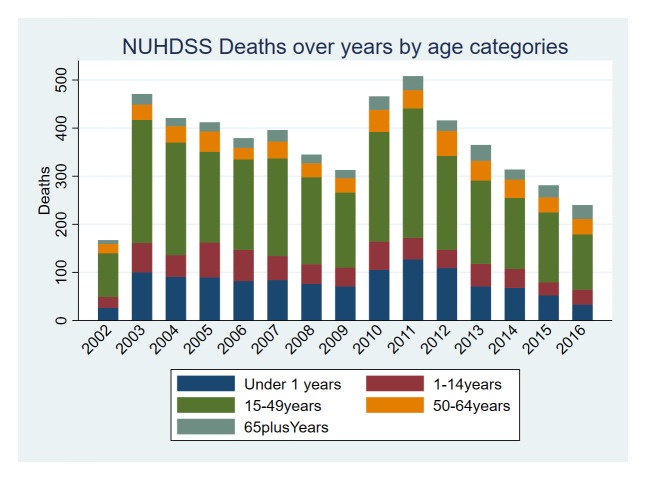
Number of deaths over years by age categories in the NUHDSS.

There were a total of 6218 deaths followed up between 2002 and 2016, of which the highest number were in 2011(533; 8.6%), out of which 5495 (88.3%) had VAs conducted. Most of the deceased were between the ages of 15–49 (3147, 50.6%). Distribution of deaths by year and age is shown in Figure 1. The most common cause of death in the period of 2002 to 2016 was tuberculosis and the least commonly identified cause of death was anaemia. The cause-specific mortality fractions are shown in Table 2.

**Table 2.** Cause-specific mortality fractions in the NUHDSS between 2002–2016.

Cause of death	CSMF ^*^
HIV/AIDS	15.60
Anaemia	0.12
Asthma	0.35
Cardiovascular	6.01
COPD ^**^	0.46
Diabetes mellitus	0.15
Diarrheal diseases	1.64
Direct obstetric	0.86
Infectious diseases	2.25
Injuries	14.07
Liver disease	0.27
Malaria	1.32
Malnutrition	0.40
Meningitis	4.22
Neonatal causes	6.30
Malignancies	3.18
Respiratory tract infections	10.24
Tuberculosis	19.14
Other communicable disease	3.14
Other non-communicable disease	2.93
Other external cause	0.61
Indeterminate	6.73
Total	100

*= Cause specific mortality fractions **= Chronic Obstructive Pulmonary Disease

### Description of VA recall time

The range of recall time for deaths with VAs done was from 1 day to 3001 days, with a median recall time of 92 days (Interquartile range 44–169 days). Majority of the verbal autopsies (45.7%) were conducted between one to 3 months of death. Distribution of socio-demographic characteristics varied based on the various verbal autopsy recall periods as shown in Table 3. There were more VAs conducted within the first month of death between 2010 and 2013 (26%) as compared to the other years under study.

**Table 3.** Distribution of background characteristics by recall time.

Recall time	< 1 month	1–3 Months	4–6 Months	7–12 Months	>12 Months	Total
	N= 5,494(100%)					
	936 (17.04)	2,509 (45.67)	1,012 (18.42)	579 (10.54)	458 (8.34)	**5,494 (100%)**
**Variable**						
**Gender**						
Female	371 (15.85)	1,071 (45.77)	457 (19.53)	247 (10.56)	194 (8.29)	2340 (100)
Male	565 (17.91)	1438 (45.59)	555 (17.60)	332 (10.53)	264 (8.37)	3154 (100)
**Age**						
<1 year	167 (14.09)	551 (46.50)	223 (18.82)	144 (12.15)	100 (8.44)	1185 (100)
1–14years	108 (15.84)	294 (43.11)	126 (18.48)	89 (13.05)	65 (9.53)	682 (100)
15–49 years	509 (18.36)	1234 (44.52)	508 (18.33)	268 (9.67)	253 (9.13)	2772 (100)
50–64 years	95 (18.16)	264 (50.48)	93 (17.78)	47 (8.99)	24 (4.59)	523 (100)
65 plus	57 (17.17)	166 (50.00)	62 (18.67)	31 (9.34)	16 (4.82)	332 (100)
**Residence**						
Korogocho	523 (16.24)	1491 (46.29)	614 (19.06)	346 (10.74)	247 (7.67)	3221 (100)
Viwandani	413 (18.18)	1018 (44.81)	397 (17.47)	233 (10.26)	211 (9.29)	2272 (100)
**Year of death**						
2002–2005	148 (10.06)	636 (43.24)	286 (19.44)	161 (10.94)	240 (16.32)	1471 (100)
2006–2009	142 (9.91)	578 (40.33)	331 (23.10)	231 (16.12)	151 (10.54)	1433 (100)
2010–2013	462 (26.32)	917 (52.25)	245 (13.96)	80 (4.56)	51 (2.91)	1755 (100)
2014–2016	184 (17.04)	378 (45.27)	150 (17.960	107 (12.81)	16 (8.34)	835 (100)
**Place of death**						
House	286 (15.04)	878 (46.19)	364 (19.15)	209 (10.99)	164 (8.63)	1901 (100)
Health facility	432 (16.88)	1162 (45.39)	479 (18.71)	271 (10.59)	216 (8.44)	2560 (100)
Enroute to health facility	65 (18.62)	150 (42.98)	63 (18.05)	44 (12.61)	27 (7.74)	349 (100)
Other	151 (22.47)	315 (46.88)	102 (15.18)	54 (8.04)	50 (7.44)	672 (100)
Missing	2 (16.67)	4 (33.33)	4 (33.33)	1 (8.33)	1 (8.33)	12 (100)
**Respondent lives in the HH**						
Yes	470 (19.00)	1118 (45.19)	441 (17.83)	272 (10.99)	173 (6.99)	2474 (100)
No	360 (18.58)	873 (45.05)	362 (18.68)	198 (10.22)	145 (7.48)	1938 (100)
Not in the universe ^*^	67 (10.29)	312 (47.93)	110 (16.90)	52 (7.99)	110 (16.90)	651 (100)
Missing	39 (9.05)	206 (47.80)	99 (22.97)	57 (13.23)	30 (6.96)	431 (100)
**Sought health care**						
Yes	662 (15.43)	1957 (45.62)	814 (18.97)	485 (11.31)	372 (8.67)	4290 (100)
No	263 (23.11)	524 (46.05)	186 (16.34)	87 (7.64)	78 (6.85)	1138 (100)
Don’t know	4 (10.53)	16 (42.11)	8 (21.05)	3 (7.89)	7 (18.42)	38 (100)
Missing	7 (25.00)	12 (42.86)	4 (14.29)	4 (14.29)	1 (8.34)	28 (100)

*Implies that the response given did not apply to the context of the deceased individual

### The effect of recall time on VA cause of death assignment

The effect of recall time varied for different causes of death ( Table 4). We found strong evidence (p-value = 0.0124) that identifying HIV/AIDS as the likely cause of death varied based on the timing of the VA. Conducting the VA 4 to 6 months after the death increased the likelihood of identifying HIV/AIDS as the cause of death by 24% compared to conducting it between 1 to 3 months (AOR 1.24; 95% CI 1.01-1.54; p-value = 0.043).

**Table 4.** Crude and adjusted logistic regression analysis to examine the effect of recall time on the VA cause of death assignment groups.

Disease/Recall time	Number (n)	OR (95% CI)	P-Value ^α^	P-value ^β^	AOR (95%CI) ^γ^	P- Value ^α^	P-Value ^δ^
**HIV/AIDS**							
<1 Month	96	0.7 (0.55-0.89)	0.004	<0.0001	0.85 (0.66-1.09)	0.198	0.0124
1–3 Months	352	Ref			Ref		
4–6 Months	178	1.31 (1.07-1.60)	0.008		1.24 (1.01-1.54)	0.043	
7–12months	99	1.26 (0.99-1.61)	0.060		1.16 (1.89-1.52)	0.256	
>12 months	63	0.98 (0.73-1.30)	0.876		0.77 (0.57-1.05)	0.106	
**Malaria -Anaemia**							
<1 Month	10	0.67 (0.33-1.34	0.254	0.3285	0.72 (0.35-1.46)	0.359	0.3606
1–3 Months	40	Ref			Ref		
4–6 Months	15	0.93 (0.51-1.69)	0.808		0.91 (0.50-1.67)	0.767	
7–12months	8	0.86 (0.40-1.86)	0.710		0.72 (0.33-1.57)	0.408	
>12 months	12	1.66 (0.86-3.19)	0.128		1.65 (0.82-3.32)	0.158	
**Tuberculosis**							
<1 Month	139	0.85 (0.70-1.06)	0.148	0.0442	0.90 (0.71-1.13)	0.381	0.7997
1–3 Months	424	Ref			Ref		
4–6 Months	182	1.08 (0.89-1.31)	0.440		1.01 (0.81-1.24)	0.963	
7–12months	89	0.89 (0.70-1.15)	0.373		0.89 (0.67-1.18)	0.437	
>12 months	96	1.30 (1.02-1.67	0.036		1.05 (0.79-1.41)	0.731	
**Respiratory tract** **infections**							
<1 Month	84	1.05 (0.81-1.37)	0.707	0.0714	1.17 (0.88-1.54)	0.280	0.0585
1–3 Months	215	Ref			Ref		
4–6 Months	95	1.11 (0.86-1.42)	0.438		1.12 (0.86-1.47)	0.380	
7–12months	62	1.28 (0.95-1.72)	0.105		1.12 (0.81-1.54)	0.493	
>12 months	58	1.54 (1.13-2.11)	0.006		1.70 (1.21-2.41)	0.002	
**Meningitis**							
<1 Month	31	0.82 (0.54-1.23)	0.333	0.4481	0.83 (0.55-1.26)	0.378	0.4409
1–3 Months	101	Ref			Ref		
4–6 Months	38	0.93 (0.64-1.36)	0.709		0.89 (0.60-1.31)	0.553	
7–12months	22	0.94 (0.59-1.51)	0.802		0.83 (0.51- 1.34	0.447	
>12 months	25	1.38 (0.88-2.16)	0.164		1.35 (0.08-2.19)	0.217	
**Other infectious** **diseases**							
<1 Month	68	1.16 (0.86-1.55)	0.329	0.0953	1.40 (1.02-1.92)	0.038	0.0214
1–3 Months	159	Ref			Ref		
4–6 Months	52	0.80 (0.58-1.10)	0.176		0.75 (0.54-1.06)	0.102	
7–12months	48	1.34 (0.95-1.87)	0.091		1.11 (0.78-1.60)	0.560	
>12 months	25	0.85 (0.55-1.32)	0.474		0.75 (0.47-1.20)	0.227	
**Maternal/neonatal**							
<1 Month	42	0.61 (0.43-0.86)	0.005	0.0618	0.57 (0.40-0.82)	0.002	0.024
1–3 Months	179	Ref			Ref		
4–6 Months	70	0.97 (0.73-1.29)	0.820		1.01 (0.75-1.36)	0.962	
7–12months	37	0.97 (0.62-1.28)	0.527		0.98 (0.67-1.43)	0.91	
>12 months	28	0.85 (0.56-1.28)	0.431		1.02 (0.66-1.58)	0.938	
**Malignancies**							
<1 Month	30	1.14 (0.74-1.75)	0.562	0.7464	1.08 (0.69-1.71)	0.726	0.5190
1–3 Months	71	Ref			Ref		
4–6 Months	24	0.83 (0.52-1.33)	0.448		0.79 (0.48-1.29)	0.340	
7–12months	17	1.04 (0.61-1.78)	0.890		1.05 (0.59-1.86)	0.871	
>12 months	16	1.24 (0.72-2.16)	0.440		1.44 (0.79-2.65)	0.228	
**Injuries/other** **external causes**							
<1 Month	221	1.62 (1.34-1.94)	<0.001	>0.001	1.28 (0.96-1.70)	0.094	0.2626
1–3 Months	403	Ref			Ref		
4–6 Months	143	0.86 (0.70 -1.06)	0.152		1.06 (0.78-1.44)	0.701	
7–12months	80	0.84 (0.65- 1.09	0.180		1.37 (0.93-2.011)	0.108	
>12 months	64	0.85 (0.64-1.13)	0.260		0.91 (0.58-1.42)	0.673	
**Cardiovascular**							
<1 Month	66	1.34 (0.99-1.82)	0.057	0.0202	1.36 (0.98-1.89)	0.064	0.0858
1–3 Months	134	Ref			Ref		
4–6 Months	64	1.19 (0.88-1.62)	0.252		1.15 (0.83-1.60)	0.394	
7–12months	24	0.77 (0.49-1.20)	0.240		0.78 (0.48-1.25)	0.299	
>12 months	16	0.64 (0.38-1.09)	0.100		0.70 (0.40-1.24)	0.226	
**Other Chronic NCDs**							
<1 Month	42	1.03 (0.72-1.49)	0.180	0.911	0.97 (0.67-1.41)	0.880	0.4877
1–3 Months	109	Ref			Ref		
4–6 Months	46	1.05 (0.74-1.49)	0.792		1.11 (0.78-1.60)	0.563	
7–12months	30	1.20 (0.79-1.82)	0.382		1.36 (0.88-2.09)	0.162	
>12 months	23	1.16 (0.73-1.85)	0.518		1.40 (0.86-2.28)	0.175	
**Indeterminate**							
<1 Month	107	0.88 (0.69-1.11)	0.268	0.0027	0.92 (0.71-1.17)	0.490	0.0339
1–3 Months	322	Ref			Ref		
4–6 Months	105	0.79 (0.62-0.99)	0.044		0.77 (0.60-0.98)	0.036	
7–12months	63	0.82 (0.62-1.10)	0.200		0.80 (0.59-1.09)	0.158	
>12 months	32	0.51 (0.35-0.74)	0.000		0.59 (0.39-0.88)	0.011	

^α^Wald p value. ^β^chi square p value ^γ^Variables adjusted in the model: Gender, Age, Slum area, Year of death, Place of death, Respondent lives in household, Sought health care ^δ^Likelihood ratio p value. OR = odds ratio, CI = confidence interval, AOR = adjusted odds ratio, Ref = reference group, HIV = human immunodeficiency virus, AIDS = acquired immunodeficiency syndrome, NCDs = non-communicable diseases.

Both the crude and the adjusted analysis showed that an assignment of malaria/anaemia, meningitis, malignancies and other chronic diseases as the probable cause of death did not depend on the timelines within which the VA was conducted. We observed that assignment of tuberculosis as the probable cause of death was dependent on the recall time in the unadjusted analysis (p value= 0.0442) but this effect was lost in the adjusted analysis.

In the crude analysis, recall time did not affect the identification of other infectious diseases as the probable cause of death but we found an effect of recall time in the adjusted analysis (p-value = 0.024). Compared to doing the VA one to three months after the death, conducting it in less than one month increased the chance of identifying other infectious diseases as the probable cause of death by 40 per cent (AOR 1.4; 95% CI 1.02-1.92). Similarly, identifying maternal/neonatal causes of death did not depend on the recall time in the crude analysis but in the adjusted analysis, there was an effect of recall time with a 43% lower chance of assigning probable cause of death to maternal/neonatal causes in VAs done less than one month compared to those done between 1 to 3 months (AOR 0.57; 95%CI 0.40-0.82; p-value=0.002). Compared to a recall period of 1–3 months, cases with recall periods of >12 months were 41% less likely to be classified as indeterminate (AOR 0.59 ; 95%CI 0.39-0.88; p value=0.01).

## Discussion

In this secondary analysis, we investigate the effect of recall time on the cause of death assigned through the InterVA-4 software. There was a notable variation in the timing of VAs with a higher percentage of VAs being conducted within three months of death in the latter years of follow up (2010 – 2016) as compared to the earlier years (2002–2009) suggesting an improved death notification and follow up system in the latter years.

The recall time in a VA is crucial as it determines if the respondent(s) can accurately recount the prevailing symptoms, signs and probable diagnosis before the deceased person’s death ^25^. The interVA-4, as well as other VA systems, rely on the accuracy of this information to assign the possible cause of death. In our adjusted analysis, we found a significant difference in the odds of the assignment of a cause of death as HIV/AIDS, other infectious disease and maternal/neonatal causes by recall time. We found that there was a 40% higher chance of identifying other infectious diseases as the cause of death in our analysis for VAs conducted less than one month of death as compared to those conducted between one to three months.‬‬‬‬‬‬‬‬‬‬‬‬‬‬‬‬‬‬‬‬‬‬‬‬‬‬‬‬‬‬‬‬‬‬‬‬‬‬‬‬‬‬‬‬‬‬‬‬‬‬‬‬‬‬‬‬‬‬‬‬‬‬‬‬‬‬‬‬‬‬‬‬‬‬‬‬‬‬‬‬‬‬‬‬‬‬‬‬‬‬‬‬‬‬‬‬‬‬‬‬‬‬‬‬‬‬‬‬‬‬‬‬‬‬‬‬‬‬‬‬‬‬‬‬‬‬‬‬‬‬‬‬‬‬‬‬‬‬‬‬‬‬‬‬‬‬‬‬‬‬‬‬‬‬‬‬‬‬‬‬

The timing of VAs should consider prevailing circumstances to the death and ensure the appropriate mourning period be observed based on the accepted cultural norms and religion. In a study ^26^ looking at effects of culture and ethics on VAs in Ghana, it was noted that the timing should take into consideration the respondent’s emotional distress, for example from the death of an only child or a maternal death. The mourning period may also vary based on the relationship between the interviewee(s) and the deceased as well as the nature of the death and role of the deceased person in the household ^26^. The Kenya 2019 verbal autopsy guidelines provide for a 30–40-day period of mourning before conducting a VA with a maximum allowable period of one year after the death ^23^.

In the NUHDSS, the VA was preceded by the field supervisor’s visit to the deceased home to determine the appropriate time to conduct the VA, based on an assessment of the situation and the availability of a credible respondent ^20^. A maximum of up to five visits were conducted to contact the deceased household members and where it was established that they were no longer residents of the area, a willing credible neighbour was interviewed. 

Some of the factors that could affected the timing of the VA are migration of the deceased family following the death due to changes in the economic situation and other factors. Mourning period’s in Kenya also vary based on tribe and cultures, with some cultures having extensive periods of mourning as compared to others, while in some cultures, the length of the mourning period will be dependent on marital status, age, gender, role in community, and birth order ^27^. Data on tribe/ethnicity was, however, not available for further analysis in this study. 

Our results, in general, find no effect of recall time on identifying the cause of death by VA for most chronic diseases (except HIV/AIDS) and indeterminate causes as compared to acute conditions. We observe that VA respondents in situations where the deceased had a chronic disease are more likely to remember the symptoms, signs and the probable cause of death overtime. 

Delay of VA for periods beyond 12 months has been hypothesised to lead to a less accurate recall of symptoms ^14^. Our analysis did not show any significant differences for any of the identified causes of deaths for recall periods above 12 months apart from the indeterminate causes of death. Compared to a recall period of 1–3 months, cases with recall periods of >12 months were 41% less likely to be classified as indeterminate. In comparison, in a study in South Africa, the only significant difference for recall periods over 12 months were for neonatal deaths ^15^.

In the regression analyses, we do not adjust for seasonality as seasons and weather patterns in Kenya have varied considerably over the period of analysis in the paper ^28^, it would be less prudent to include seasonality in the model. Additionally, the NUHDSS is located in an urban informal settlement where disruptions of participant availability for reasons like farming are less as compared to a similar study done in South Africa ^15^

The interVA-4 outputs consist of three likelihoods, other approaches to the analysis include inclusion of all the possible causes of death and their likelihoods as weights to the regression models. However, where there is need to recategorize the causes of the death, assumptions will have to be made on how the likelihoods combine before and after the categorisation, whether additive, multiplicative or other complex form of combination. We avoid these assumptions by conducting the regression analysis with the most probable cause of death as assigned by the interVA-4. We acknowledge that there might me alternative preferences to this.

The ideal method of identifying the effect of recall time would have been a comparison of the cause of death generated from gold standard death certification to the VA assigned cause of death for the different recall times. However, in the absence of the gold standard comparator, as in our case, the alternative approach used in this paper was to compare probabilities or odds of making a specific VA cause of death assignment for different recall periods. To our best knowledge this is the first paper within the East African region to expound on the effects of recall time in VAs. 

## Conclusion

Recall time affected the assignment of VA cause of death for HIV/AIDS, other infectious diseases, maternal /neonatal and indeterminate causes. Our analysis indicates that in the urban informal setting, VAs should be conducted from one month up to 6 months after the death to improve the probability of accurately assigning the cause of death.

## Data availability

The KENYA - NUHDSS - Verbal Autopsy, Causes of deaths 2002–2015 dataset was used in this secondary analysis. The dataset is available upon request from the APHRC Microdata Portal http://microdataportal.aphrc.org/index.php/catalog. Access to the dataset can be obtained through submission of a written request in the APHRC portal following creation of an account. Further information on how to apply for access to the data can be found here.
