# Supplementary material for: Does recall time matter in verbal autopsies? Evidence from urban informal settlements in Nairobi, Kenya
Source: Wellcome Open Res. 2021 Mar 1;5:217. Originally published 2020 Sep 16. [Version 2] doi: 10.12688/wellcomeopenres.16243.2 (PMC8030111; doi:10.12688/wellcomeopenres.16243.2)
Supplement: Peer review history

# Peer review: Reviewer response for version 2

**DOI:** 10.21956/wellcomeopenres.18326.r42886

**Published:** 2021-04-06

**Authors:** Lucia D'Ambruoso

**Affiliations:** 1 University of Aberdeen, Aberdeen, UK; 2 University of the Witwatersrand, Johannesburg, South Africa

Overall comments:

 Thank you for the opportunity to review the revised paper, which appears to have responded to comments on the earlier version. This is an interesting paper considering recall time as a key a methodological aspect of the VA method. The final, published piece might usefully be checked for grammar.

Is the work clearly and accurately presented and does it cite the current literature?

Yes

If applicable, is the statistical analysis and its interpretation appropriate?

I cannot comment. A qualified statistician is required.

Are all the source data underlying the results available to ensure full reproducibility?

Partly

Is the study design appropriate and is the work technically sound?

Partly

Are the conclusions drawn adequately supported by the results?

Partly

Are sufficient details of methods and analysis provided to allow replication by others?

Partly

Reviewer Expertise:

Global public health; qualitative methods; health policy and systems research; South Africa

I confirm that I have read this submission and believe that I have an appropriate level of expertise to confirm that it is of an acceptable scientific standard.

# Peer review: Reviewer response for version 2

**DOI:** 10.21956/wellcomeopenres.18326.r42887

**Published:** 2021-03-22

**Authors:** Aaron S. Karat

**Affiliations:** 1 TB Centre, London School of Hygiene & Tropical Medicine, London, UK

My thanks to the authors for their careful and detailed responses and my apologies for the delay in re-reviewing. I am happy with the changes made and have no further concerns - congratulations on the paper!

Is the work clearly and accurately presented and does it cite the current literature?

Partly

If applicable, is the statistical analysis and its interpretation appropriate?

Partly

Are all the source data underlying the results available to ensure full reproducibility?

Yes

Is the study design appropriate and is the work technically sound?

Partly

Are the conclusions drawn adequately supported by the results?

Partly

Are sufficient details of methods and analysis provided to allow replication by others?

Yes

Reviewer Expertise:

Tuberculosis, epidemiology, infectious diseases, verbal autopsy.

I confirm that I have read this submission and believe that I have an appropriate level of expertise to confirm that it is of an acceptable scientific standard.

# Response

**Published:** 2021-03-30

**Authors:** Donnie Mategula

**Affiliations:** Malawi-Liverpool Wellcome Trust Clinical Research Programme, Malawi

Thank you for the review and the kind words.

# Peer review: Reviewer response for version 1

**DOI:** 10.21956/wellcomeopenres.17841.r41360

**Published:** 2020-12-08

**Authors:** Aaron S. Karat

**Affiliations:** 1 TB Centre, London School of Hygiene & Tropical Medicine, London, UK

Thank you for the opportunity to review this paper, which is generally well written and deals with an important aspect of verbal autopsy implementation. This is a secondary analysis of VA data from more than 5,000 deaths and, though the findings are interesting, I have some concerns around some of the methods and around the interpretation of results.

**Major comments:**

1. Calculation of CSMFs - The method used to calculate CSMFs is potentially problematic. InterVA output (up to three causes with likelihoods +/- residual likelihood) is intended to be converted into CSMFs in a way that takes account of all causes of death assigned (see Appendix 4 of the InterVA-4 user guide), not only the most likely cause, as described by the authors. The ‘collapsing’ process described seems perfectly justifiable but may have been better applied after the calculation of CSMFs to reflect better the true output of InterVA. Adopting this approach would likely have produced different CSMFs for each recall period, and may therefore have affected the results of the regression.
2. Logistic regression - Although most factors likely to affect the relationship between recall time and cause of death were adjusted for, time of year/seasonality was not. This is potentially very important, as it may have affected mortality patterns but also affected the ability of VA teams to access respondents (e.g. this may have been harder in the rainy season) and therefore affected time between death and VA. This may be less relevant in the urban setting where this study was conducted (compared with the rural setting in the study by Hussain-Alkhateeb *et al.*) but this should at least have been discussed and the omission justified by the authors.
3. Interpretation of results - My apologies if I have misunderstood, but I do not entirely follow some of the statements made in the discussion and I am not sure the conclusions flow from the results presented. The authors suggest that respondents may be more likely to remember the symptoms of an acute illness if the VA is done in a shorter time after death, yet in this analysis the odds of being assigned a respiratory tract infection CoD were higher for VAs done after >12 months compared with 1–3 months.
4. I also do not entirely follow the possible explanation for why maternal deaths were less likely to be assigned <1 months after death than 1–3 months. Are the authors suggesting that fewer maternal deaths were assigned because VAs may have been delayed when a particular cause of death was suspected? It would seem important to know if this was done when any other cause of death was suspected, or if it was only for maternal deaths. The authors also state that the Kenyan guidelines recommend 30–40 days mourning prior to conducting a VA, but there is no description of how often these guidelines were followed (and/or if they were followed more closely in one district than another) – this is a potentially a major source of bias and should ideally have been explored in more detail in the analysis.

**Minor comments:**

**Introduction: **

1. **Paragraph 1:** ****The ‘gold standard’ for cause of death is one assigned by complete diagnostic autopsy, not by a medical practitioner, though of course for the majority of deaths a practitioner-assigned CoD is often the best that can be hoped for. It may be worth making this point a little more clearly in the first paragraph.
2. **Paragraph 2: **This is a very minor point, but the multiple plurals used here (‘interviews’, ‘questionnaires’, ‘relatives’, ‘caregivers’) suggests that a standard VA involves interviewing more than one person, when this is rarely the case.
3. **Paragraph 2:** I believe the full name of the organisation is the Population Health Metrics Research Consortium. A better reference when discussing the VA instrument (instead of the paper currently cited in Ref 7) may be the ‘VA tools’ page on the PHMRC website.
4. **Paragraph 3:** the list of CCVA methods is slightly dated (the ref provided is a 2014 review). This pre-print may provide a more up-to-date list (InterVA is also misspelled in this paragraph, as “inter-VA”).

**Methods:**

1. If possible, please could the authors provide the exact cut-offs (in days) used for the recall time categories. At present it is not entirely clear if a VA conducted after ~3.5 months would be categorised as “1–3 months” or “4–6 months” (same for recall times between 6 and 7 months).
2. There may be a good reason for using 1–3 months as a reference instead of <1 month but the reason for this approach is not described in the methods. In my opinion this makes it more difficult to interpret the results and to look at trends across recall periods (regardless of statistical ‘significance’).

**Results**

1. **Page 4:** it may be more interesting to show the median + IQR for recall time, given that it is unlikely to be normally distributed?
2. **Table 2:** It is not clear what ‘Not in the universe’ means (under ‘Respondent lives in the HH’).
3. **Table 2:** Typo under ‘year of death (“2104”).
4. **Table 3:** It would make the results more transparent if the CSMF proportion for each cause was shown in this table, very ideally for each recall period for each cause, so readers are clear on the ‘number’ of outcomes associated with each OR/aOR.
5. **Table 3:** Typo in the 95% CI for the aOR for HIV/AIDS deaths with a recall time of 7–12 months (“10.89”).

Is the work clearly and accurately presented and does it cite the current literature?

Partly

If applicable, is the statistical analysis and its interpretation appropriate?

Partly

Are all the source data underlying the results available to ensure full reproducibility?

Yes

Is the study design appropriate and is the work technically sound?

Partly

Are the conclusions drawn adequately supported by the results?

Partly

Are sufficient details of methods and analysis provided to allow replication by others?

Yes

Reviewer Expertise:

Tuberculosis, epidemiology, infectious diseases, verbal autopsy.

I confirm that I have read this submission and believe that I have an appropriate level of expertise to confirm that it is of an acceptable scientific standard, however I have significant reservations, as outlined above.

# Response

**Published:** 2021-02-03

**Authors:** Donnie Mategula

**Affiliations:** Malawi-Liverpool Wellcome Trust Clinical Research Programme, Malawi

**Dr Aaron S.Karat** Many thanks for your helpful and thoughtful comments. We have re-submitted an updated manuscript which addresses your suggestions and concerns. Please find our detailed responses below.

Major comments:

 1) Calculation of CSMFs - The method used to calculate CSMFs is potentially problematic. Inter-VA output (up to three causes with likelihoods +/- residual likelihood) is intended to be converted into CSMFs in a way that takes account of all causes of death assigned (see Appendix 4 of the InterVA-4 user guide), not only the most likely cause, as described by the authors. The ‘collapsing’ process described seems perfectly justifiable but may have been better applied after the calculation of CSMFs to reflect better the true output of InterVA. Adopting this approach would likely have produced different CSMFs for each recall period, and may therefore have affected the results of the regression.

**Response: **Thank you for the comment. The cause specific mortality fractions have been re-calculated as suggested and are presented in table 2 of the revised paper. However, we have not used the CSMFs for the regression analysis as adding the cause of death specific weights/likelihoods to a model where the cause of death have been collapsed makes assumptions on how the cause of death weights/likelihoods combine before and after the re-categorization. To avoid making these assumptions, we prefer to use the most likely cause of death in the logistic regression analysis. However, we have included this limitation in the discussion.

 2) Logistic regression - Although most factors likely to affect the relationship between recall time and cause of death were adjusted for, time of year/seasonality was not. This is potentially very important, as it may have affected mortality patterns but also affected the ability of VA teams to access respondents (e.g. this may have been harder in the rainy season) and therefore affected time between death and VA. This may be less relevant in the urban setting where this study was conducted (compared with the rural setting in the study by Hussain-Alkhateeb *et al.*) but this should at least have been discussed and the omission justified by the authors.

**Response: **Seasons  and weather patterns in Nairobi have varied considerably over the period of analysis in the paper, it would have been less prudent to include seasonality in the model. Additionally , we agree with the reviewer that this variable is  less relevant in this context unlike in the Hussain Alkhateeb et al paper. We have included text in the discussion section to reflect our rationale for the lack of inclusion of this variable in the model.

 3) Interpretation of results - My apologies if I have misunderstood, but I do not entirely follow some of the statements made in the discussion and I am not sure the conclusions flow from the results presented. The authors suggest that respondents may be more likely to remember the symptoms of an acute illness if the VA is done in a shorter time after death, yet in this analysis the odds of being assigned a respiratory tract infection CoD were higher for VAs done after >12 months compared with 1–3 months.

**Response: **In our analysis, there is no overall association between  recall time and RTI based on the likelihood ratio test (P value 0.0585) and that is why we did not focus on RTIs. Our general observation that respondents are more likely to remember acute illness if the VA is done in a shorter time still holds. 

 4) I also do not entirely follow the possible explanation for why maternal deaths were less likely to be assigned <1 months after death than 1–3 months. Are the authors suggesting that fewer maternal deaths were assigned because VAs may have been delayed when a particular cause of death was suspected? It would seem important to know if this was done when any other cause of death was suspected, or if it was only for maternal deaths. The authors also state that the Kenyan guidelines recommend 30–40 days mourning prior to conducting a VA, but there is no description of how often these guidelines were followed (and/or if they were followed more closely in one district than another) – this is a potentially a major source of bias and should ideally have been explored in more detail in the analysis.

**Response: **Thank you for the comment. We have updated the discussion section to further explain the potential sources of delay to conducting a VA in the NUHDSS. This was not specific to maternal deaths but to the prevailing circumstances. We have edited the discussion to focus less on the maternal/neonatal finding. While the numbers for combined direct obstetric and neonatal causes were low, the majority of these were neonatal deaths. The effect observed could therefore have  been  a chance finding due to the few numbers or as a result of the higher proportion of neonatal deaths to direct obstetric causes ( 6 to 1) especially in cases where the neonatal deaths were not reviewed promptly.

Minor comments:

 Introduction: 

 5) Paragraph 1: The ‘gold standard’ for cause of death is one assigned by complete diagnostic autopsy, not by a medical practitioner, though of course for the majority of deaths a practitioner-assigned CoD is often the best that can be hoped for. It may be worth making this point a little more clearly in the first paragraph.

**Response: **This has been updated

****

 6)Paragraph 2: This is a very minor point, but the multiple plurals used here (‘interviews’, ‘questionnaires’, ‘relatives’, ‘caregivers’) suggests that a standard VA involves interviewing more than one person, when this is rarely the case.

**Response: **Multiple plurals have been reviewed and revised

 7) Paragraph 2: I believe the full name of the organisation is the Population Health Metrics Research Consortium. A better reference when discussing the VA instrument (instead of the paper currently cited in Ref 7) may be the ‘VA tools’ page on the PHMRC website.

**Response: **The changes have been made and the reference updated

****

 8) Paragraph 3: the list of CCVA methods is slightly dated (the ref provided is a 2014 review). This pre-print may provide a more up-to-date list (InterVA is also misspelled in this paragraph, as “inter-VA”).

**Response: **We  have updated the list of CCVA methods based on the updated reference, however we have retained our original citation as well since Mapundo et al  paper is in a preprint.

 Methods:

 9) If possible, please could the authors provide the exact cut-offs (in days) used for the recall time categories. At present it is not entirely clear if a VA conducted after ~3.5 months would be categorised as “1–3 months” or “4–6 months” (same for recall times between 6 and 7 months).

**Response: **Thank you for the comment,   the highest cutoff integer month for our categories included all the possible decimals before the next category, for example 1-3 months includes all possible days up to 3.99.We have updated this in the methods section.

 10) There may be a good reason for using 1–3 months as a reference instead of <1 month but the reason for this approach is not described in the methods. In my opinion this makes it more difficult to interpret the results and to look at trends across recall periods (regardless of statistical ‘significance’).

**Response: **The 1 to 3 months recall time was used as the reference category for the logistic regression for two reasons; the highest number of VAs were conducted within this period and secondly this falls within the Kenya VA guidelines on appropriate time to conduct a VA thus we set to compare all the results against this recall period. We have updated this in the methods section.

 Results

 11) Page 4: it may be more interesting to show the median + IQR for recall time, given that it is unlikely to be normally distributed?

**Response: **Thanks, we concur. The Median and IQR for recall time have been included.

****

 12) Table 2: It is not clear what ‘Not in the universe’ means (under ‘Respondent lives in the HH’).

**Response: **Implies that the response given did not apply to the context of the deceased under question. We have added a footnote in  the table to explain

 13) Table 2: Typo under ‘year of death (“2104”).

**Response: **This has been amended, thank you

 14) Table 3: It would make the results more transparent if the CSMF proportion for each cause was shown in this table, very ideally for each recall period for each cause, so readers are clear on the ‘number’ of outcomes associated with each OR/aOR.

**Response: **We have added the number of outcomes associated with each OR/aOR. Please also refer to our response to comment number 1 to justify our choice.

****

 14 Table 3: Typo in the 95% CI for the aOR for HIV/AIDS deaths with a recall time of 7–12 months (“10.89”).

**Response: **This has been amended, thank you

# Peer review: Reviewer response for version 1

**DOI:** 10.21956/wellcomeopenres.17841.r41357

**Published:** 2020-12-02

**Authors:** Lucia D'Ambruoso

**Affiliations:** 1 University of Aberdeen, Aberdeen, UK; 2 University of the Witwatersrand, Johannesburg, South Africa

**Overall comments:**

 This is an interesting paper examining recall times on the outcomes of VA surveys and contributes to the evidence base on this methodology. The paper is generally well written and clearly presented. Overall comments are as follows: first, the paper would be improved with fuller justification of key assumptions and aspects of the analytical approach around ideal recall times and CoD categories (see specific comments). Second, while I am not well-qualified to comment on statistical aspects, it would appear that not all the statistically significant findings are considered in the narrative. Third, the discussion could be further developed by considering the plausibility of the main findings, offering reasons as to why the recall time effects on some cause of death categories were found. Fourth, further attention to the social and cultural aspects of recall time, and in the context of Covid-19/other infectious outbreaks would add to the current relevance of the paper. Finally, a statistician should review the paper. As a qualitative researcher, I have not commented on the statistical approach in anything more than general terms. The paper is likely to be worthy of indexing, with attention to these issues.

**Specific comments:**

Introduction:

1. Paragraph 3: the authors may wish to acknowledge that computer-coded VA addresses observed inconsistencies within and between physician coders, as well as obviating the time implications of physician coding: in the settings where VA is needed, using physician time for VA interpretation is ethically problematic.
2. Paragraph 4: the need to account for social and cultural factors as a key factor determining recall period is convincingly introduced – how the authors deal with this issue is not clear however and could usefully be expanded on in the paper.

Methods:

1. Paragraph 1, Study setting: A map would be good to include, if possible.
2. Paragraph 3, Design: The term demographic health system may be misleading, suggest replace and use HDSS consistently.
3. Paragraph 3, Design: InterVA5 model is currently available, it may be worth considering applying InterVA5 for these data.
4. Page 4, Table 1: the title of this table might be rephrased - strictly speaking, criteria are not presented. Recategorization of InterVA CoDs to constructed CoD categories might be more accurate.
5. Page 4, Table 1: The collapsed CoD categories are not quite clear. The authors state that category classifications were guided by counts and reasonable relatedness - this requires some clarification, especially around amenia and malaria. The authors also state that the collapsed categories are based on classifications used in other work, however there are 8 categories in the Hussain *et al.* study and 15 in the Oti and Kyobutungi paper. The relationship of these to the 12 categories used in this analysis similarly would benefit from some clarification.
6. Page 4, Table 1: It is not customary to cite AIDS/HIV as a category, rather HIV/AIDS, as is done later in the paper – suggest consistency with commonly used term.
7. Page 4, paragraph 1: CSMFs typically account for all probable CoD assigned per case. Suggest recalculating the CSMF using all probable CoDs from InterVA.
8. It is not entirely clear why 1-3 months recall time is used as the reference period. The paper indeed presents research from the same region where 12 months recall is found to be suitable. The authors may wish to clarify this element of the analysis.

Results:

- While I am not well-qualified to comment on the statistical analyses, it would appear that not all significant findings from the adjusted regression analysis are highlighted in the narrative, please see (d) and (e) below. The main findings would appear to be as follows: Compared to a recall period of 1-3 months.
  - (a) Cases with recall periods of 4-6 months are 24% more likely to be classified as due to HIV;
  - (b) Cases with recall periods of <1 month 40% more likely to be classified as due to other infectious causes.
  - (c) Cases with recall periods of <1 month 43% less likely to be classified as due to maternal causes.
  - (d) Cases with recall periods of >12 months 70% more likely to be classified as due to RTI.
  - (e) Cases with recall periods of >12 months 41% less likely to be classified as indeterminate.

Discussion:

1. The discussion could be further developed by considering the plausibility of the main findings offering reasons as to why the recall time effects on some cause of death categories were found. For example, that cases with recall periods of <1 month are 43% less likely to be classified as due to maternal causes is counter-intuitive – maternal deaths are relatively rare, generally avoidable and therefore likely to be highly memorable.
2. Page 9, paragraph 5: the statement ‘our analysis did not show any significant differences for any of the identified causes of deaths for recall periods above 12 months’ needs to be checked. Again, from a general perspective, Table 3 appears to suggest that deaths investigated >12 months are (a) 70% more likely to be classified as due to RTI (AOR 1.70 (CI 1.21-2.41) P=0.002) and (b) 41% less likely to be diagnosed by InterVA as indeterminate (AOR 0.59 (CI 0.39-0.88) P=0.011).
3. Further attention to social and cultural influences on recall times, and how these vary by country/region would be useful in this section. Thorough assessment of social and cultural norms prior to VA surveys is of critical importance, whether these factors have an impact on the findings presented in this paper would be useful to consider.
4. Some attention to how the analysis and recommendations on recall periods related to the Covid-19 pandemic and other infectious outbreaks, e.g., Ebola, is likely to be of relevance to readers.
5. The authors may wish to include strengths and limitations section, where a sensitivity analysis with e.g., up to 12 months as an ideal recall period (as is indicated in other research) is applied.

Is the work clearly and accurately presented and does it cite the current literature?

Yes

If applicable, is the statistical analysis and its interpretation appropriate?

I cannot comment. A qualified statistician is required.

Are all the source data underlying the results available to ensure full reproducibility?

Partly

Is the study design appropriate and is the work technically sound?

Partly

Are the conclusions drawn adequately supported by the results?

Partly

Are sufficient details of methods and analysis provided to allow replication by others?

Partly

Reviewer Expertise:

Global public health; qualitative methods; health policy and systems research; South Africa

I confirm that I have read this submission and believe that I have an appropriate level of expertise to confirm that it is of an acceptable scientific standard, however I have significant reservations, as outlined above.

# Response

**Published:** 2021-02-03

**Authors:** Donnie Mategula

**Affiliations:** Malawi-Liverpool Wellcome Trust Clinical Research Programme, Malawi

**Dr Lucia D'Ambruoso, **Many thanks for your helpful and thoughtful comments. We have re-submitted an updated manuscript which addresses your suggestions and concerns. Please find our detailed responses below:

Introduction:

 Paragraph 3: the authors may wish to acknowledge that computer-coded VA addresses observed inconsistencies within and between physician coders, as well as obviating the time implications of physician coding: in the settings where VA is needed, using physician time for VA interpretation is ethically problematic.

**Response: **Thank you for the suggestion. This has been included.

 Paragraph 4: the need to account for social and cultural factors as a key factor determining recall period is convincingly introduced – how the authors deal with this issue is not clear however and could usefully be expanded on in the paper.

**Response: **We have considered the social factors that were available in the dataset in the regression analysis. In the discussion, we have expounded on some of the potential social-cultural factors around recall period not available in the dataset.

Methods:

 Paragraph 1, Study setting: A map would be good to include, if possible.

 A reference to where the NUHDSS map can be accessed has been provided in the revised version.

 Paragraph 3, Design: The term demographic health system may be misleading, suggest replace and use HDSS consistently.

**Response: **This has been updated.

 Paragraph 3, Design: InterVA5 model is currently available, it may be worth considering applying InterVA5 for these data.

 Thank you for the comment.  The data set was provided following the Inter-VA4 analysis therefore it would not be possible to analyze it using Inter-VA5. InterVA-5 was developed to accommodate the WHO 2016 standard VA tool that was not utilized for this data set.

 Page 4, Table 1: the title of this table might be rephrased - strictly speaking, criteria are not presented. Recategorization of InterVA CoDs to constructed CoD categories might be more accurate.

**Response: **Thank you. This has been updated

 Page 4, Table 1: The collapsed CoD categories are not quite clear. The authors state that category classifications were guided by counts and reasonable relatedness - this requires some clarification, especially around amenia and malaria. The authors also state that the collapsed categories are based on classifications used in other work, however there are 8 categories in the Hussain *et al.* study and 15 in the Oti and Kyobutungi paper. The relationship of these to the 12 categories used in this analysis similarly would benefit from some clarification.

**Response: **We have updated the text to further detail how we did the recategorization. The approach to this classification has been used by other studies as cited, however the final number of categories varies depending on data and specific circumstances in the study areas. The collapsing of malaria and anemia is based on the precinct that malaria is a major cause of anemia in tropical areas.

 Page 4, Table 1: It is not customary to cite AIDS/HIV as a category, rather HIV/AIDS, as is done later in the paper – suggest consistency with commonly used term.

 This has been updated. Thank you.

 Page 4, paragraph 1: CSMFs typically account for all probable CoD assigned per case. Suggest recalculating the CSMF using all probable CoDs from InterVA.

 CSMFs have been recalculated as suggested.It is not entirely clear why 1-3 months recall time is used as the reference period. The paper indeed presents research from the same region where 12 months recall is found to be suitable. The authors may wish to clarify this element of the analysis.

**Response: **The 1 to 3 months recall time was used as the reference category for the logistic regression for two reasons; the highest number of VAs were conducted within this period and secondly this falls within the Kenya VA guidelines on appropriate time to conduct a VA thus we set to compare all the results against this recall period. We have updated this in the methods section.

Results:

 While I am not well-qualified to comment on the statistical analyses, it would appear that not all significant findings from the adjusted regression analysis are highlighted in the narrative, please see (d) and (e) below. The main findings would appear to be as follows: Compared to a recall period of 1-3 months.

 (a) Cases with recall periods of 4-6 months are 24% more likely to be classified as due to HIV;

 (b) Cases with recall periods of <1 month 40% more likely to be classified as due to other infectious causes.

 (c) Cases with recall periods of <1 month 43% less likely to be classified as due to maternal causes.

 (d) Cases with recall periods of >12 months 70% more likely to be classified as due to RTI.

 (e) Cases with recall periods of >12 months 41% less likely to be classified as indeterminate.

**Response: **Thank you. We have updated the missing significant results for the indeterminate, however we have left out the RTIs as the association with recall time is not significant on the overall likelihood ratio test of association.

Discussion:

 The discussion could be further developed by considering the plausibility of the main findings offering reasons as to why the recall time effects on some cause of death categories were found. For example, that cases with recall periods of <1 month are 43% less likely to be classified as due to maternal causes is counter-intuitive – maternal deaths are relatively rare, generally avoidable and therefore likely to be highly memorable.

**Response: **While the numbers for combined direct obstetric and neonatal causes were low, the majority of these were neonatal deaths. The effect observed could therefore have  been  a chance finding due to the few numbers or as a result of the higher proportion of neonatal deaths to direct obstetric causes ( 6 to 1) especially in cases where the neonatal deaths were not reviewed promptly.

 Page 9, paragraph 5: the statement ‘our analysis did not show any significant differences for any of the identified causes of deaths for recall periods above 12 months’ needs to be checked. Again, from a general perspective, Table 3 appears to suggest that deaths investigated >12 months are (a) 70% more likely to be classified as due to RTI (AOR 1.70 (CI 1.21-2.41) P=0.002) and (b) 41% less likely to be diagnosed by InterVA as indeterminate (AOR 0.59 (CI 0.39-0.88) P=0.011).

**Response: **Thank you for the observation. We have revised the statement and included the findings on the indeterminate causes of death. In our analysis, there is no  evidence of an overall association between  recall time and RTI based on the likelihood ratio test (P value 0.0585) and that is why we did not focus on RTIs in the results section

 Further attention to social and cultural influences on recall times, and how these vary by country/region would be useful in this section. Thorough assessment of social and cultural norms prior to VA surveys is of critical importance, whether these factors have an impact on the findings presented in this paper would be useful to consider.

**Response:** Thank you for the suggestion. We have included an assessment of the potential social and cultural circumstances prior to a VA.

 Some attention to how the analysis and recommendations on recall periods related to the Covid-19 pandemic and other infectious outbreaks, e.g., Ebola, is likely to be of relevance to readers.

**Response: **Thank you for the suggestion, however, given the period within which the VA were conducted (2002-2016) it might be difficult to extrapolate the findings to the current situation of Covid-19.
